# Cardiopulmonary Fitness and Exercise‐Based Cardiac Rehabilitation in Patients With Myocarditis

**DOI:** 10.1155/cdr/3611934

**Published:** 2026-10-12

**Authors:** Si-Si Zhang, Lu-Ying Jiang, Hou-Juan Zuo, Wan-Jun Liu

**Affiliations:** ^1^ Department of Cardiology Beijing Anzhen Hospital National Clinical Research Center for Cardiovascular Diseases Capital Medical University Beijing China ccmu.edu.cn; ^2^ Division of Cardiology Department of Internal Medicine Tongji Medical College Tongji Hospital Huazhong University of Science and Technology Wuhan China hust.edu.cn

**Keywords:** cardiac rehabilitation, cardiopulmonary fitness, fulminant myocarditis, nonfulminant

## Abstract

**Background:**

Cardiorespiratory fitness (CRF) is a powerful predictor of cardiovascular morbidity and mortality. The cardiopulmonary exercise test (CPET) is considered a gold standard in assessing CRF. Patients with myocarditis are recommended to abstain from exercise because of the risk of sudden cardiac death (SCD). Therefore, the clinical benefit of exercise‐based cardiac rehabilitation (CR) has not been previously established in this population.

**Objective:**

The aim of this study is to assess the CRF of myocarditis and the safety and effectiveness of an exercise‐based CR program in these populations.

**Method:**

A total of 70 consecutive patients with a diagnosis of myocarditis admitted to Tongji Hospital, Tongji Medical College, Huazhong University of Science and Technology between October 2021 and December 2024 were enrolled in the current study. CPET was performed within 3–6 months of hospital discharge. The exercise‐based CR program was performed for 3 months. The protocol included biomarker analysis, CPET, and standard‐of‐care echocardiography (including resting left ventricular ejection fraction and global longitudinal strain [GLS]). Data were collected at admission and 3 months after discharge.

**Results:**

A total of 70 patients with myocarditis were enrolled. Among them, 47 patients completed the rehabilitation program and had complete follow‐up assessments, including cardiopulmonary exercise testing, and were included in the final analysis. In comparison with NFM, patients with FM were more often female, and had higher levels of creatine kinase (CK), lactate dehydrogenase (LDH), urea nitrogen (UN), N‐terminus probrain natriuretic peptide (NT‐proBNP), fasting blood glucose (FBG), and white blood cell (WBC). For FM, echocardiography revealed more frequently signs of left ventricular (LV) dysfunction in the form of a reduced ejection fraction (*p* < 0.01), and lower GLS (*p* < 0.01) in contrast to NFM. CPET results showed that FM patients had markedly lower exercise capacity, impaired ventilatory efficiency, and pulmonary function (*p* < 0.05). After the 3‐month exercise‐based CR program, both groups demonstrated significant improvements in peak VO_2_, peak workload, and MVV. However, no significant between‐group differences were observed in the magnitude of improvement in CRF.

**Conclusion:**

CRF was substantially impaired in patients recovering from myocarditis, particularly among those with fulminant presentations. Participation in a structured 3‐month exercise‐based CR program was feasible and was associated with favorable changes in exercise capacity and cardiopulmonary performance.

**Trial Registration:** Chinese Clinical Trial Registry: ChiCTR2400088436

## 1. Introduction

Myocarditis is defined as an inflammatory damage of the myocardium, which is most often caused by viral infection or autoimmune disease [1, 2]. According to the clinical symptoms/signs, echocardiographic, hemodynamic, and histological findings, myocarditis can be categorized as nonfulminant myocarditis (NFM) or fulminant myocarditis (FM) [2, 3]. FM is defined as acute myocarditis with rapid‐onset severe hemodynamic compromise which will require high doses of vasoactive drugs or mechanical circulatory support (MCS) including extracorporeal membrane oxygenation (ECMO), intra‐aortic balloon pump (IABP), or ventricular assist devices (VAD). NFM refers to clinically confirmed myocarditis without hemodynamic collapse or need for advanced circulatory support. FM may develop fatal ventricular arrhythmia, rapid and severe hemodynamic instability, cardiogenic shock, and even sudden death [1, 4].

Although multidisciplinary cardiac rehabilitation (CR) is effectively used in patients with cardiovascular disease, there are no studies that expand the evidence of CR also being clinically beneficial for patients with myocarditis [5].

The Sport Cardiology Section of the European Associationof Preventive Cardiology (EAPC) recommended that athletes with myocarditis should be restricted from exercise programs for a period of 3–6 months, according to the clinical severity and duration of the illness, left ventricle (LV) function at onset, andextent of inflammation on the CMR [6]. Moreover, because of the scarcity of data, the healthcare professionals are often cautious, even hesitant to offer exercise prescriptions for myocarditis populations.

As a result, few studies address exercise training for patients with myocarditis in clinical practice.

The cardiopulmonary exercise test (CPET) is considered the gold standard for the assessment of exercise capacity, cardiorespiratory fitness, encompassing respiratory and metabolic response to exercise, which is widely applied in several clinical practices [7–9]. Despite this, the application of CPET to the myocarditis populations has been limited.

The aims of the present study were as follows: (1) to assess the value of the CRF of myocarditis populations and (2) to evaluate the effects of the exercise‐based CR program and its safety.

## 2. Method

Between December 2022 and October 2024, patients with myocarditis were recruited from the Tongji Hospital, Tongji Medical College, and Huazhong University of Science and Technology. The diagnosis of myocarditis was made based on clinical symptoms (chest pain, dyspnoea, and syncope), laboratory investigation (elevated high‐sensitivity troponin, B‐type natriuretic peptide [BNP], and markers of inflammation), cardiac magnetic resonance (CMR) Lake Louise criteria (hyperaemia, tissue oedema, and necrosis/fibrosis) [10, 11], and/or the endomyocardial biopsy (EMB). Dallas criteria with associated immunohisto‐chemical and molecular criteria [12]. Detailed etiological classification of myocarditis was not consistently available for all patients. Therefore, patients were classified according to clinical presentation (fulminant vs. NFM) rather than underlying etiology. Etiological data were not included in the present analysis. Patients were eligible for inclusion if they met all of the following criteria: age between 18 and 70 years; clinically stable for at least 3 months after hospital discharge, with no evidence of active inflammation or hemodynamic instability; left ventricular ejection fraction (LVEF) ≥ 40%.

The exclusion criteria of the study were LVEF < 40% or New York Heart Association (NYHA) Classes III–IV symptoms; ongoing myocardial inflammation as confirmed by CMR imaging or elevated cardiac biomarkers; severe arrhythmias (e.g., sustained ventricular tachycardia, high‐grade atrioventricular block) or presence of an implanted cardioverter‐defibrillator; musculoskeletal, pulmonary, or neurological disorders limiting exercise testing; pregnancy or lactation.

### 2.1. CPET

All participants completed the symptom‐constrained maximum load exercise test on a cycle ergometer using the ramped Bruce protocol. The protocol began with a 2‐min unloaded warm‐up (0 W), followed by continuous ramp‐wise workload increments of 5–20 W per minute, depending on the participant′s clinical status at the speed of 55–65 cycles/min, until the following symptoms or signs were observed: inability to maintain pedaling rate more than 50 rpm/min; ST‐segment depression (> 2 mm); abnormal blood pressure response (SBP > 250 mmHg or DBP > 120 mmHg); the respiratory exchange ratio (RER) ≥ 1.10, or self‐reported rating of perceived exertion (RPE) ≥8 [13–15]. Breath‐by‐breath gas analysis, heart rate (HR), oxygen saturation, blood pressure, and ECG were continuously measured throughout the test. Cardiopulmonary fitness and pulmonary function including peak oxygen uptake (peak VO_2_), oxygen uptake of anaerobic threshold (AT VO_2_), RER, peak workload, peak metabolic equivalents (METs), peak METs, peak HR, O_2_ pulse, slope of ventilatory equivalent for carbon dioxide (VE/VCO_2_), ΔVO_2_/Δwork power (ΔVO_2_/ΔWR), forced expiratory volume in the first second (FEV1), forced vital capacity (FVC), the FEV1/FVC ratio, maximum vital capacity (VCmax), and the maximum voluntary ventilation (MVV).

### 2.2. Echocardiography

Transthoracic echocardiography was performed in accordance with current guidelines [16]. Two‐dimensional images were acquired in parasternal long‐axis (PLAX), short‐axis (PSAX), apical four‐chamber, and two‐chamber views. The following parameters were recorded: the size of the LV, the early diastolic inflow velocity (E), velocity during active atrial contraction (A), the E to A wave (E/A) ratio, and ejection fraction (EF), which was calculated by modified Simpson′s rule.

Left ventricular speckle tracking imaging was performed semiautomatically in the three apical views (apical four‐chamber, apical two‐chamber, and apical three‐chamber) at the end of expiration in three consecutive cardiac cycles with a temporal resolution of 60–90 frames/s. The LV was divided into 17 segments, and the average peak systolic longitudinal strain of all segments was calculated to obtain global longitudinal strain (GLS). The normal of GLS was defined as > 18% [16].

### 2.3. Blood Sampling

Blood samples were collected after 8–12 h of fasting. Fasting blood glucose (FBG), total cholesterol (TC), triglyceride (TG), high‐density lipoprotein (HDL), low‐density lipoprotein (LDL), lipoprotein (a) (Lp(a)), lactate dehydrogenase (LDH), creatinine (Cr), urea nitrogen (UN), uric acid (UA), troponin, creatine kinase (CK), high sensitivity C‐reactive protein (hsCRP), erythrocyte sedimentation rate (ESR), N‐terminus probrain natriuretic peptide (NT‐proBNP), interleukin‐1*β* (IL‐1*β*), interleukin‐2 (IL‐2), interleukin‐6 (IL‐6), interleukin‐8 (IL‐8), interleukin‐10 (IL‐10), tumor necrosis factor‐*α* (TNF‐*α*), procalcitonin (PCT), red blood cell (RBC), white blood cell (WBC), blood platelet, and hemoglobin (Hb) were measured and collected.

### 2.4. Exercise‐Based CR Prescription

At 3 months after discharge, participants returned to the hospital and completed the CPET to evaluate the CPF. In cases where clinical risk is deemed excessively high, or when CPET results indicate that exercise training is not suitable, we recommend a re‐evaluation of the exercise program following the implementation of optimal medical management. The exercise programs were individualized and consistent with international recommendations with the so‐called FITT‐VP model (frequency, intensity, time, and type) [17, 18]. The exercise program was supervised by a sports physician or a nurse, which consisted of 30–40 min/day and 3–5 days/week of aerobic exercise lasting for 12 weeks. The intensity of aerobic exercise was set based on the heart rate (HR) at AT obtained during the CPET. The target HR was set close to the HR at the anaerobic threshold [19].

### 2.5. Statistical Analysis

Continuous variables were presented as mean ± SD, and the categorical variables were reported as absolute numbers or percentages. Two‐sample *t*‐tests or the Mann–Whitney *U* test were used to compare the difference between the two populations. The chi‐square test was used for categorical variables. All tests were two‐sided, and significance was set at a *p* value < 0.05. Statistical analyses were conducted using SPSS Version 21.0 (IBM).

## 3. Results

A total of 70 patients were initially screened and enrolled. Only patients with complete baseline and follow‐up clinical and cardiopulmonary exercise testing data were included in the final analysis. Consequently, 47 patients met the criteria for complete evaluation and constituted the study cohort. The baseline characteristics are outlined in Table 1. The two populations were similar for most variables, except for a higher frequency of males in the NFM population. FM patients displayed higher levels of CK, LDH, UN, NT‐proBNP, FBG, and WBC compared with NFM (*p* < 0.05, respectively). Additionally, the FM group exhibited a longer median hospital stay of 10 days versus 6.00 days (*p* < 0.001). There were no significant differences in relation to inflammatory biomarkers between the two groups.

**Table 1:** Population characteristics.

Characteristic	FM (*n* = 27)	NFM (*n* = 20)	*p*
Male sex, *n* (%)	14 (51.9)	16 (80)	0.04
Age, mean (SD), *y*	37 ± 19.87	30.4 ± 12.66	0.172
BMI, (kg/m^2^)	23.06 ± 3.64	23.7 ± 3.05	0.525
Length of hospital stay (d)	10 (8, 12)	6 (4.15,8.75)	0.001
Medications			
*β*‐blocker	18 (66.7)	17 (85)	0.154
ACEI/ARB	20 (74.1)	12 (60)	0.306
Trimetazidine	25 (92.6)	19 (95)	0.739
Laboratory data			
Troponin (pg/mL)	19331.7 (1460.6, 43814.4)	4351. (1695.3, 20145.7)	0.106
CK (U/L)	661.5 (293.5, 1109.5)	254 (126, 265)	0.032
LDH (U/L)	428 (248, 681)	241 (185.5, 318.5)	0.012
hsCRP (mg/L)	17.8 (4.5, 56.1)	10.6 (2.45, 79.5)	0.576
UN (mmol/L)	5.31 ± 1.98	4.19 ± 1.19	0.031
Cr (umol/L)	66 (56, 81)	71.5 (63, 79.5)	0.628
UA (umol/L)	326.71 ± 106.1	325.82 ± 97.33	0.977
ESR (mm/H)	6.5 (3.75, 19.25)	6.5 (3.25, 12)	0.806
NT‐proBNP (pg/mL)	3993 (1463.5, 8513)	874.8 (192.5, 2349.5)	0.002
FBG (mmol/L)	6.5 (5.62, 8.11)	5.59 (5.29, 6.87)	0.028
TC (mmol/L)	3.43 ± 1.02	3.47 ± 0.75	0.894
TG (mmol/L)	0.97 (0.77, 1.55)	0.82 (0.65, 1.19)	0.278
HDL (mmol/L)	0.94 ± 0.27	0.97 ± 0.23	0.671
LDL (mmol/L)	2.25 ± 0.96	2.18 ± 0.61	0.779
Lp(a) (mmol/L)	30.7 (13.2,68.7)	17.1 (11.9, 28.8)	0.15
PCT(ng/ml)	0.13 (0.05, 0.39)	0.05 (0.05, 0.18)	0.064
TNF‐*α* (pg/mL)	8.6 (6.4, 12.5)	11.6 (6.9, 19)	0.26
IL‐1*β* (pg/mL)	5 (5, 11)	5 (5, 13)	0.63
IL‐2 (U/mL)	599 (306, 815)	338 (244, 509)	0.078
IL‐6 (pg/mL)	9.62 (5.2, 26.9)	10.27 (3.57, 32.62)	0.871
IL‐8 (pg/mL)	17.1 (7.1, 38.1)	20.6 (11.1, 122)	0.305
IL‐10 (pg/mL)	5 (5, 9.2)	5 (5, 5)	0.148
Hb (g/L)	129.74 ± 26.19	139.5 ± 16.09	0.528
RBC (×10^12^/L)	4.59 ± 0.69	4.66 ± 0.48	0.736
WBC (×10^9^/L)	9.27 (6.73, 12.5)	8.81 (6.26, 9.57)	0.046
Blood platelet (×10^9^/L)	215.08 ± 75.63	220.3 ± 46.08	0.788

Abbreviations: ACEI, Angiotensin‐converting enzyme inhibitor; ARB, Angiotensin II receptor blocker; CK, creatine kinase; Cr, creatinine; BMI, body mass index; ESR, erythrocyte sedimentation rate; FBG, fasting blood glucose; Hb, hemoglobin; HDL, high‐density lipoprotein; hsCRP, high sensitivity C‐reactive protein; IL‐1*β*, interleukin‐1*β*; IL‐2, interleukin‐2; IL‐6, interleukin‐6; IL‐8, interleukin‐8; IL‐10, interleukin‐10; LDH, lactate dehydrogenase; LDL, low‐density lipoprotein; Lp(a), Lipoprotein (a); NT‐proBNP, N‐terminus probrain natriuretic peptide; PCT, procalcitonin; RBC, red blood cell; TC, total cholesterol; TG, triglyceride; TNF‐*α*, tumor necrosis factor‐*α*; UA, uric acid; UN, urea nitrogen; WBC, white blood cell.

At the time of enrollment in the CR program, no patients had an implantable cardioverter‐defibrillator (ICD), and no patients were using a wearable cardioverter‐defibrillator (WCD, LifeVest). All participants underwent comprehensive clinical evaluation before rehabilitation initiation. Patients with active myocarditis, hemodynamic instability, uncontrolled ventricular arrhythmias, or other contraindications to exercise training were excluded from participation. Therefore, the study population represented clinically stable myocarditis survivors deemed suitable for exercise‐based CR.

The length of hospital stay differed substantially according to disease severity. Patients with FM generally experienced longer hospitalizations than those with NFM, reflecting the greater need for intensive care, hemodynamic support, and prolonged clinical recovery during the acute phase of the disease.

Echocardiographic appearances are presented in Table 2. At baseline, the patients with FM showed significantly lower LVEF [0.44 (0.31–0.50) vs. 0.57 (0.48–0.60), *p* = 0.003], lower early diastolic mitral annular velocity (e ^′^) [6 (3.75–10.25) vs. 11 (6–13) cm/s, *p* = 0.007], and more impaired GLS (−11.27*%* ± 4.79*%* vs. −16.39*%* ± 3.76*%*, *p* = 0.001) compared with those with NFM. No significant differences were observed in other echocardiographic parameters between the two groups.

**Table 2:** Echocardiographic features of patients with myocarditis.

Variables	FM (*n* = 27)	NFM (*n* = 20)	*p*
LV EDD, mm	45.69 ± 6.22	46.55 ± 3.81	0.568
LA diameter, mm	32.81 ± 6.95	34.85 ± 5.74	0.293
LV ejection fraction, %	0.44 (0.31, 0.5)	0.57 (0.48, 0.6)	0.003
E wave, cm/s	76 ± 24.93	83.93 ± 17.64	0.295
A wave, cm/s	49 (38.25, 74.25)	47 (39, 58)	0.889
E/A ratio	1.62 ± 0.82	1.76 ± 0.57	0.59
e ^′^, cm/s	6 (3.75, 10.25)	11 (6, 13)	0.007
a ^′^, cm/s	6.95 ± 2.64	8.13 ± 1.74	0.132
GLS (%)	−11.27 ± 4.79	−16.39 ± 3.76	0.001

Abbreviations: EDD, indicates end‐diastolic diameter; GLS, global longitudinal strain; LA, left atrial; LV, left ventricular.

### 3.1. CPET Analysis

Patients in the FM group demonstrated significantly lower exercise capacity compared with the NFM group. Peak VO_2_, peak workload, peak METs, and peak O_2_ pulse were all significantly reduced, whereas VO_2_ at anaerobic threshold did not differ significantly. Ventilatory efficiency was impaired in the FM group, as reflected by a higher VE/VCO_2_ slope, and slightly higher *Δ*VO_2_/*Δ*WR (*p* < 0.05). In addition, pulmonary function was also reduced in FM patients. FEV_1_, VCmax, and MVV were all significantly lower, whereas FEV_1_/FVC ratio did not differ (Table 3).

**Table 3:** Cardiopulmonary exercise testing parameters in patients with myocarditis precardiac and postcardiac rehabilitation.

Variables	FM (*n* = 27)		*p*	NFM (*n* = 20)		*p*	Δ *p*value between groups
	Pre	Post		Pre	Post		
Peak VO_2_ (mL/min)	17.88 ± 3.93	19.89 ± 5.95	0.041	21.13 ± 4.19^a^	23.51 ± 4.99	0.009	0.784
Peak load (w)	83.81 ± 31.08	93.74 ± 28.77	0.005	109.75 ± 22.86^a^	121.35 ± 20.46	0.012	0.317
Peak Mets	5.19 ± 1.2	5.67 ± 1.69	0.116	6.03 ± 1.21^a^	6.73 ± 1.43	0.018	0.601
VO_2_ at AT (mL/min)	11.96 ± 2.31	12.03 ± 3	0.873	12.74 ± 2.66	13.84 ± 3.23	0.038	0.139
Peak VE (mL/kg/min)	589.1 ± 117	635.74 ± 191.9	0.275	606.78 ± 157.15^a^	705.16 ± 174.24	0.038	0.406
VE/VCO_2_ slope	25.74 ± 4.4	18.11 ± 5.02	0.02	21.85 ± 4.16	22.33 ± 4.62	0.586	0.156
DeltaVO_2_/deltaWR	10.46 (9.2, 12)	11.77 (10.35, 14.14)	0.003	11.41 (10.83, 14.32)^a^	11.38 (10.52, 13.18)	0.131	0.355
Peak RER	1.17 ± 0.11	1.17 ± 0.09	0.9	1.17 ± 0.11	1.23 ± 0.11	0.1	0.219
Peak HR (bmp/min)	147.41 ± 23.63	136.78 ± 30.42	0.094	141.35 ± 17.25	151.95 ± 16.85	0.05	0.01
Peak O_2_ pulse	8.1 ± 2.98	9.35 ± 2.7	0.037	10.51 ± 2.24^a^	10.82 ± 2.06	0.386	0.349
FEV1 (L)	2.45 ± 0.79	2.53 ± 0.81	0.928	3.06 ± 0.89^a^	3.16 ± 0.58	0.531	0.327
FVC (L)	3.2 ± 0.97	3.2 ± 0.97	0.531	3.85 ± 0.63^a^	3.85 ± 0.63	0.063	0.072
FEV1/FVC	0.79 (0.7, 0.84)	0.83 (0.73, 0.91)	0.253	0.84 (0.65, 0.89)	0.84 (0.77, 0.89)	0.422	0.317
VCmax (L)	3.33 ± 0.96	3.3 ± 0.98	0.668	4.08 ± 0.73^a^	3.98 ± 0.67	0.233	0.228
MVV (L)	94.32 ± 36.29	112.4 ± 39.74	0.001	123.84 ± 29.03^a^	139.63 ± 30.55	0.012	0.807

Abbreviations: AT, anaerobic threshold; bpm, beats per minute; FEV1, forced expiratory volume in the first second; FVC, forced vital capacity; HR, heart rate; METs, metabolic equivalent; MVV, maximum voluntary ventilation; RER, respiratory exchange ratio; VC, vital capacity; VCO_2_, exhaled carbon dioxide; VE, minute ventilation; VO_2_, oxygen uptake; WR, work rate.

^a^Pre FM vs pre NFM *p* < 0.05.

### 3.2. Exercise‐Based CR Effects

Overall, in both groups combined, 3‐month exercise‐based training was associated with *a* 2.16 ± 4.53 mL/kg/min increase in peak VO_2_ with substantial individual‐level variability in the change of exercise performance indices compared with their baseline levels, including peak load, peak Mets, peak VE, *Δ*VO_2_/*Δ*WR, VE/VCO_2_, Peak O_2_plus, and MVV (Table 4, Figure 1).

**Table 4:** Cardiopulmonary exercise testing parameters in patients with myocarditis precardiac and postcardiac rehabilitation.

Variables	FM (*n* = 27)		*p*	NFM (*n* = 20)		*p*	Δ *p*value between groups
	Pre	Post		Pre	Post		
Peak VO_2_ (mL/min)	17.88 ± 3.93	19.89 ± 5.95	0.041	21.13 ± 4.19^a^	23.51 ± 4.99	0.009	0.784
Peak load (w)	83.81 ± 31.08	93.74 ± 28.77	0.005	109.75 ± 22.86^a^	121.35 ± 20.46	0.012	0.317
Peak Mets	5.19 ± 1.2	5.67 ± 1.69	0.116	6.03 ± 1.21^a^	6.73 ± 1.43	0.018	0.601
VO_2_ at AT (mL/min)	11.96 ± 2.31	12.03 ± 3	0.873	12.74 ± 2.66	13.84 ± 3.23	0.038	0.139
Peak VE (mL/kg/min)	589.1 ± 117	635.74 ± 191.9	0.275	606.78 ± 157.15^a^	705.16 ± 174.24	0.038	0.406
VE/VCO_2_ slope	25.74 ± 4.4	18.11 ± 5.02	0.02	21.85 ± 4.16	22.33 ± 4.62	0.586	0.156
DeltaVO_2_/deltaWR	10.46 (9.2, 12)	11.77 (10.35, 14.14)	0.003	11.41 (10.83, 14.32)^a^	11.38 (10.52, 13.18)	0.131	0.355
Peak RER	1.17 ± 0.11	1.17 ± 0.09	0.9	1.17 ± 0.11	1.23 ± 0.11	0.1	0.219
Peak HR (bmp/min)	147.41 ± 23.63	136.78 ± 30.42	0.094	141.35 ± 17.25	151.95 ± 16.85	0.05	0.01
Peak O_2_ pulse	8.1 ± 2.98	9.35 ± 2.7	0.037	10.51 ± 2.24^a^	10.82 ± 2.06	0.386	0.349
FEV1 (L)	2.45 ± 0.79	2.53 ± 0.81	0.928	3.06 ± 0.89^a^	3.16 ± 0.58	0.531	0.327
FVC (L)	3.2 ± 0.97	3.2 ± 0.97	0.531	3.85 ± 0.63^a^	3.85 ± 0.63	0.063	0.072
FEV1/FVC	0.79 (0.7, 0.84)	0.83 (0.73, 0.91)	0.253	0.84 (0.65, 0.89)	0.84 (0.77, 0.89)	0.422	0.317
VCmax (L)	3.33 ± 0.96	3.3 ± 0.98	0.668	4.08 ± 0.73^a^	3.98 ± 0.67	0.233	0.228
MVV (L)	94.32 ± 36.29	112.4 ± 39.74	0.001	123.84 ± 29.03^a^	139.63 ± 30.55	0.012	0.807

Abbreviations: AT, anaerobic threshold; bpm, beats per minute; FEV1, forced expiratory volume in the first second; FVC, forced vital capacity; HR, heart rate; METs, metabolic equivalent; MVV, maximum voluntary ventilation; RER, respiratory exchange ratio; VC, vital capacity; VCO_2_, exhaled carbon dioxide; VE, minute ventilation; VO_2_, oxygen uptake; WR, work rate.

^a^Pre FM vs pre NFM *p* < 0.05.

**Figure 1:**
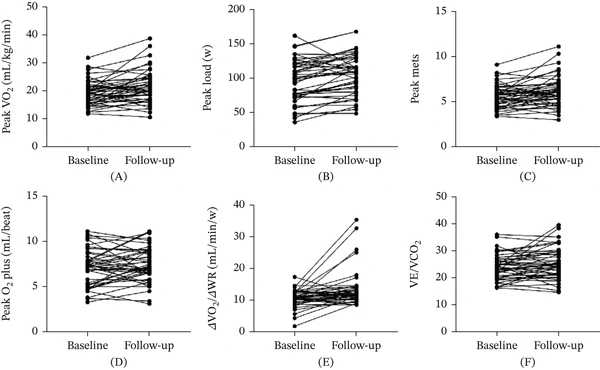
Individual changes in cardiopulmonary exercise testing parameters before and after cardiac rehabilitation.

Among AM subtypes, FM groups also showed significant improvement regarding the VE/VCO_2_ slope, *Δ*VO₂/*Δ*WR, peak HR, and peak O_2_ pulse. However, we found no statistically significant between‐group difference for changes in the CRF after the CR program (Table 3, Figure 2).

**Figure 2:**
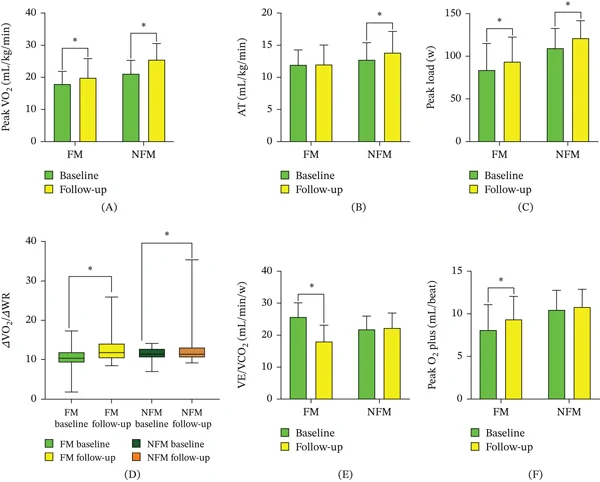
Changes in cardiopulmonary exercise testing parameters after 3 months of cardiac rehabilitation in patients with fulminant and nonfulminant myocarditis.

## 4. Discussion

Previous data are scarce about the cardiopulmonary status of AM, especially for FM. CRF has not been well established in myocarditis. Current guidelines recommend a 3–6 months of exercise restrictions with a slow and cautious return to activity for the myocarditis population [20, 6].

The primary aim of this study was to evaluate the overall effects of exercise‐based CR in a real‐world myocarditis population, whereas subgroup comparisons between FM and NFM were performed to provide additional descriptive clinical context.

In this study, we analyzed the clinical, echocardiographic, and cardiopulmonary characteristics of patients with AM. The major new finding of this study is the following: (1) patients with FM exhibited significantly impaired exercise capacity, pulmonary function, and ventilatory efficiency compared with those with NFM; (2) exercise‐based training can safe and lead to functional improvement for AM population; and (3) exercise‐related improvement in VO_2_peak did not differ between AM subtypes (FM and NFM).

The observed impairment in exercise capacity and ventilatory efficiency among patients with FM may be explained by several potential mechanisms. First, extensive myocardial inflammation and necrosis during the acute phase of FM can lead to persistent subclinical myocardial injury; lack of contractile reserve may contribute to decreased cardiac output, with a corresponding decrease in peak VO_2_ [10]. Second, autonomic dysfunction and abnormal peripheral chemoreflex sensitivity following severe inflammatory stress may contribute to an exaggerated ventilatory response, thereby increasing the VE/VCO_2_ slope and reducing ventilatory efficiency [21]. Third, pulmonary involvement and respiratory muscle deconditioning may play an important role, as suggested by the reduced FEV_1_, VCmax, and MVV with a preserved FEV_1_/FVC ratio, indicating a predominantly restrictive or deconditioning‐related ventilatory limitation rather than airway obstruction. In addition, prolonged immobilization, systemic inflammation, and catabolic stress during the acute illness phase may lead to skeletal muscle dysfunction, mitochondrial impairment, and reduced peripheral oxygen utilization, further contributing to exercise intolerance and an abnormal *Δ*VO_2_/*Δ*WR relationship [22, 23]. In chronic HF, low peak VO_2_peak is known to identify patients at higher mortality risk, with a peak VO_2_ of 14 mL/kg/min selected as a criterion for acceptance for cardiac transplant [24, 25]. Beyond risk stratification, CPET‐derived parameters also provide integrated insights into cardiovascular, pulmonary, and peripheral determinants of exercise intolerance. Although myocarditis differs pathophysiologically from chronic heart failure, the present findings suggest that similar principles may apply, as patients with FM exhibited markedly reduced peak VO_2_ and other indices of exercise performance. This observation highlights the potential clinical value of CPET in characterizing functional impairment and recovery in myocarditis.

In our study, a 3‐month exercise‐based training program was associated with a modest but clinically meaningful improvement in cardiorespiratory fitness when both groups were analyzed together, as reflected by an average increase of 2.16 ± 4.53 mL·kg^−1^·min^−1^ in peak VO_2_. This improvement was accompanied by substantial interindividual variability across multiple exercise performance indices, including peak workload, peak METs, peak VE, *Δ*VO_2_/*Δ*WR, VE/VCO_2_ slope, peak O_2_ pulse, and MVV, highlighting the heterogeneous physiological response to exercise training in patients with acute myocarditis. Notably, patients with FM also demonstrated significant improvements in several CPET‐derived parameters, particularly ventilatory efficiency (VE/VCO_2_ slope), metabolic efficiency (*Δ*VO_2_/*Δ*WR), chronotropic response (peak HR), and stroke volume surrogate (peak O_2_ pulse), suggesting that structured exercise training may partially reverse abnormalities in ventilatory control and cardiovascular reserve even in this more severe phenotype. Despite these within‐group improvements, no statistically significant between‐group differences were observed in changes in cardiorespiratory fitness following the CR program. This finding may indicate a comparable trainability of patients with FM and NFM once clinical stability is achieved, although the relatively small sample size and substantial interindividual variability may have limited the statistical power to detect group‐level differences. Collectively, these results support the feasibility and potential benefit of exercise‐based CR in patients recovering from acute myocarditis, while also emphasizing the need for individualized training prescriptions and larger prospective studies to better define responders, optimal training intensity, and long‐term functional and prognostic effects.

Although no patients in our cohort required ICD implantation or WCD therapy during the rehabilitation period, arrhythmic risk remains an important consideration in patients recovering from myocarditis. Ventricular arrhythmias and sudden cardiac death may occur in a subset of patients, particularly those with persistent left ventricular dysfunction, extensive late gadolinium enhancement on CMR imaging, or a history of sustained ventricular arrhythmias. Therefore, comprehensive arrhythmic risk stratification should be performed before initiating exercise‐based CR.

Importantly, no professional or competitive athletes were included in this study, which enhances the internal consistency of the cohort and avoids confounding effects related to pre‐existing high cardiorespiratory fitness. Although myocarditis in athletes is an important clinical scenario, our findings primarily reflect outcomes in a general clinical population referred for rehabilitation after recovery from myocarditis.

## 5. Limitations

Several limitations of this study should be acknowledged. First, this was a single‐center study with a relatively small sample size, which may limit the generalizability of our findings and reduce the statistical power to detect between‐group differences in training‐related changes, particularly between FM and NFM; therefore, these comparisons should be interpreted as exploratory and hypothesis‐generating rather than confirmatory. Given the relatively small sample size, particularly in the subgroup analyses, the present study was primarily intended to explore potential associations and generate hypotheses rather than provide precise effect estimates. Larger studies will be needed to more accurately quantify the magnitude of rehabilitation‐related changes. Another limitation is the lack of comprehensive etiological data. Because definitive etiological classification was unavailable for a substantial proportion of patients, we were unable to assess whether specific causes of myocarditis influenced exercise capacity, recovery trajectories, or response to CR. Future studies incorporating systematic etiological evaluation may provide further insights into the relationship between myocarditis etiology and rehabilitation outcomes. Second, the duration of follow‐up was limited to 3 months, and thus, the long‐term sustainability of functional improvements and their association with clinical outcomes such as recurrence, hospitalization, or prognosis could not be evaluated. Third, selection bias cannot be excluded, as only clinically stable patients who were able to undergo CPET and participate in exercise training were included, potentially underrepresenting more severe or unstable cases. Fourth, although multiple CPET‐derived parameters were analyzed, invasive hemodynamic measurements and direct assessments of peripheral muscle or autonomic function were not available, limiting mechanistic interpretation. In addition, the absence of a control group of myocarditis patients who did not undergo CR; consequently, the observed improvements in cardiopulmonary fitness cannot be attributed exclusively to the rehabilitation intervention. Some degree of recovery may reflect the natural course of myocardial healing and functional restoration following myocarditis. Therefore, our findings should be interpreted as demonstrating an association between participation in CR and improved exercise capacity rather than establishing a causal effect. Prospective controlled studies are warranted to further define the independent contribution of CR to recovery after myocarditis.

## 6. Conclusion

In conclusion, patients with acute myocarditis, particularly those with fulminant presentations, exhibit significant impairments in exercise capacity, ventilatory efficiency, and pulmonary function despite clinical stabilization. Participation in an exercise‐based CR program was associated with improvements in cardiopulmonary fitness and functional capacity among clinically stable patients recovering from myocarditis. These findings support the feasibility and potential value of CR in this population; however, controlled prospective studies are needed to confirm its efficacy and establish causality.

## Author Contributions

Si‐Si Zhang and Lu‐ying Jiang were responsible for conceptualizing and designing the study. Data collection and acquisition were carried out by Lu‐ying Jiang. Data analysis, interpretation, and statistical procedures were performed by Si‐Si Zhang. The initial manuscript draft was written and revised by Si‐Si Zhang, with further revision support provided by Hou‐Juan Zuo. Hou‐Juan Zuo and Wan‐Jun Liu contributed equally to this work.

## Funding

No funding was received for this manuscript.

## Ethics Statement

The study was performed according to the declaration of Helsinki, approved by the ethical committee of Tongji Hospital (TJ‐IRB20231152). All patients provided written informed consent.

## Conflicts of Interest

The authors declare no conflicts of interest.

## Data Availability

The data that support the findings of this study are available from the corresponding authors upon reasonable request.
